# Vascular Analysis of Type 1, 2, and 3 Macular Neovascularization in Age-Related Macular Degeneration Using Swept-Source Optical Coherence Tomography Angiography Shows New Insights into Differences of Pathologic Vasculature and May Lead to a More Personalized Understanding

**DOI:** 10.3390/biomedicines10030694

**Published:** 2022-03-17

**Authors:** Henrik Faatz, Kai Rothaus, Martin Ziegler, Marius Book, Britta Heimes-Bussmann, Daniel Pauleikhoff, Albrecht Lommatzsch

**Affiliations:** 1 Department of Ophthalmology, St. Franziskus Hospital, 48145 Muenster, Germany; kai.rothaus@augen-franziskus.de (K.R.); martin.ziegler@augen-franziskus.de (M.Z.); marius.book@augen-franziskus.de (M.B.); britta.heimes@augen-franziskus.de (B.H.-B.); dapauleikhoff@muenster.de (D.P.); albrecht.lommatzsch@augen-franziskus.de (A.L.); 2 Achim Wessing Institute for Imaging in Ophthalmology, University of Essen-Duisburg, 45147 Essen, Germany; 3 Department of Ophthalmology, University of Essen-Duisburg, 45147 Essen, Germany

**Keywords:** MNV morphology, imaging, age-related macular degeneration, OCT angiography, choroidal neovascularization

## Abstract

Background: The clinical appearance of macular neovascularization (MNV) in age-related macular degeneration (nAMD) varies widely, but so far, this has had no relevance in terms of therapeutic approaches or prognosis. Therefore, our purpose was to investigate if and which differences exist in the vascular architecture of MNV and to quantify them. Methods: In 90 patients with newly diagnosed nAMD, MNV was identified by means of optical coherence tomography angiography (OCTA), and automated quantitative vascular analysis was carried out. The analyzed vascular parameters were area, flow, fractal dimension (FD), total vascular length (sumL), number of vascular nodes (numN), flow, and average vessel caliber (avgW). The current classification of MNVs divides them according to their localization into type 1 (grown from the choroid below the RPE), type 2 (grown from the choroid through RPE), and type 3 (grown from the retina toward the RPE). We compared the analyzed vascular parameters of each of the three MNV types. Kruskal–Wallis test was applied, Dunn test was performed for post hoc analysis, and for pairwise comparison, *p*-values were adjusted using Bonferroni comparison. Results: Regarding the MNV area, there was no significant difference between types 1 and 2, but type 3 was significantly smaller than types 1 and 2 (*p* < 0.00001). For FD, types 1 and 2 did not differ significantly, but again, type 3 was lower than type 1 and 2 (*p* < 0.00001). The numN were significantly higher in types 1 and 3 than in 2 (*p* < 0.005), but not between types 1 and 3. No significant differences were found between MNV types for flow. As for sumL, types 1 and 2 did not differ significantly, but type 3 was significantly lower than types 1 and 2 (*p* < 0.00001). For avgW, there was no significant difference between types 1 and 2 or between types 2 and 3, but type 3 was significantly larger than type 1 (*p* < 0.05). Conclusions OCTA yields detailed information on the vascular morphology of MNV in patients with nAMD and is able to show differences among types 1, 2, and 3. Especially comparing types 1 and 2 with type 3 reveals significant differences in area, FD, sumL, and numN. One explanation could be the similar pathogenesis of types 1 and 2 with their origin in the choroid and their growth towards the retinal pigment epithelium (RPE), whereas type 3 originates in the deep capillary plexus. Between types 1 and 2, however, only the numN differ significantly, which could be due to the fact that type 1 spreads horizontally below the RPE and, thus, display more vascular branching, while type 2 grows more vertically through the RPE and under the neurosensory retina. Detailed information about the pathologic vasculature is important for proper monitoring of the disease and to assess the efficacy of medication, especially with regard to new substances. This should be taken into consideration in future studies.

## 1. Introduction

Age-related macular degeneration (AMD) is a progressive disease that can develop macular neovascularization (MNV) in advanced stages and is referred to as neovascular AMD (nAMD) [1]. It is a multifactorial disease for which aging is the most important risk factor. The RPE phagocytes the photoreceptor cells and, through lysosomes, degrades them intracellularly to lipofuscin. The lipofuscin accumulates over time in the RPE cells leading to toxic reactions and finally to cell death. The induction of reactive oxygen species, which are specially formed under exposure to blue light (390–550 nm), is considered a key factor in the resulting retinal degeneration, as RPE cells, photoreceptors, pericytes, and endothelial cells, in particular, react with degeneration and apoptosis [2,3]. The triggered oxidative stress and the consecutive inflammation also lead to the release of angiogenic factors [4]. Since anti-vascular endothelial growth factor (VEGF) agents represent an effective therapy for MNV, the adequate diagnosis of nAMD is of enormous importance for the further course of the disease.

Fluorescence angiography (FA) with indocyanine green (ICG) is the gold standard for the diagnosis of nAMD. FA involves the intravenous administration of fluorescein to detect vascular abnormalities of the retina and choroid in the shape of hypo- or hyper fluorescence [1]. Precise analysis of vascular morphology on FA is not possible due to leakage from the MNV. Optical coherence tomography angiography (OCTA), however, detects the movement of red blood cells and, thus, offers a new perspective on physiological and pathological blood flow in the retina and choroid. It permits detailed visualization and analysis of the vascular architecture of MNV.

MNVs are divided into three subtypes according to their localization: In Type 1, MNV vessels from the choriocapillaris grow into the sub-RPE space. Type 2 MNVs also originate from the choriocapillaris, but vessels penetrate the RPE and spread subretinally. Type 3 MNVs comprise proliferative vessels that extend from the deep capillary plexus of the retina towards the outer retina. Late-stage type 3 MNVs break through Bruch’s membrane, causing pigment epithelial detachments (PED) and choroidal neovascularizations (CNV). The exact differentiation of MNV types is not always possible using present imaging modalities as in OCT vessels are not visible, localization of MNV can only be estimated, and in FA leakage phenomena obscure the vascular structure.

The Consensus Nomenclature for Reporting Neovascular Age-Related Macular Degeneration Study Group [5] emphasizes the importance of OCTA as an additional imaging modality for establishing a more detailed classification of nAMD. The current classification system takes into account the subjective appearance of an MNV and its location in relation to different retinal segments, but not the detailed vascular architecture. Anti-VEGF therapy directly targets the pathologic vasculature of MNV. Therefore, an imaging modality that allows direct visualization of the vascular morphology is most adequate for disease monitoring. It might also enable physicians to identify new pathologic features and perhaps to create an advanced classification. However, this demands the correlation of vascular parameters to clinical data, which should be done in future studies. Numerous studies have depicted the vascular morphology of MNV. In this context, the MNV has been described subjectively according to its shape (medusa, seafan, glomerulus), branching, anastomoses, and loops, as well as according to feeder vessel and halo signs [6,7,8,9], or has been quantitively analyzed in terms of its area, flow, fractal dimension, branching pattern and vessel length [10,11,12]. Specific vascular patterns of MNV have also been identified as being relevant biomarkers. For example, Cabral et al. have shown that the presence of an immature blood flow pattern on OCTA is associated with a lower rate of progression of macular atrophy [13]. Ahmed et al. investigated the vascular architecture of the MNV in OCTA with regard to its shape, branching pattern, anastomoses, loops, feeder vessel, and halo signs and found a high correlation of these characteristics to the active and inactive stages [7]. Furthermore, it could be shown that FD is higher in the active stage of MNV than in the inactive stage, which suggests a more complex vascular structure and, thus, the therapeutic effect of anti-VEGF agents on MNV could mathematically be classified [12,14,15].

The pathophysiology between the different MNV types is not yet fully understood; known differences are origin, localization, morphological changes of the retina, and clinical presentation. Moreover, we expect differences in vascular architecture between the MNV types. As nAMD is a vascular disease and OCTA allows detailed analysis of the pathologic vasculature of MNV, we hope to gain new insights into the disease through vascular analyses. Since the introduction of OCTA, subclinical MNVs that do not show exudation have also been detected [16]. These findings might also influence future therapeutic approaches, as they show that more information is needed to clarify which patient benefits most from what kind of therapy and to improve efficacy by developing new drugs. To date, it has not been possible to illustrate when and why MNV remains confined to the sub-RPE space (type 1 MNV) or grows through the RPE (type 2 MNV). It is also unclear what leads to the exudation of MNV into the different retinal compartments.

The aim of this study is to explore the differences of pathologic vasculature among untreated MNV of types 1, 2, and 3 with regard to further differentiation and quantification.

## 2. Methods

The study was conducted according to the tenets of the Declaration of Helsinki and was approved by the ethics committee of the Westphalia–Lippe Medical Association and the University of Münster (No. 2021-066-f-S). All participating patients have given their written consent. Data acquisition was retrospective with consecutive patient inclusion.

### 2.1. Study Population

Treatment-naïve nAMD patients were included in the study. General data such as age, sex, BCVA, and history of other ocular diseases were collected. In all patients, nAMD was initially diagnosed by means of FA, SD-OCT (Spectralis© HRA+OCT, Heidelberg Engineering, Heidelberg, Germany), and clinical examination. The diagnosis was verified by a senior grader at the reading center of M^3^ Macula Monitor Münster based upon the characteristic appearance of the different MNV types in multimodal imaging [5,7].

### 2.2. OCTA Imaging Analysis

All patients underwent OCTA using the swept-source OCTA PLEX^®^ Elite 9000 device (Carl Zeiss Meditec, Dublin, CA, USA), which, applying a wavelength of 1060 nm and 100,000 A-scans/s in a 6 × 6-mm scan, records two consecutive B-scans with 500 A-scans each. Automatic suppression of artifacts was selected because this improves visualization of MNV [17]. For evaluation, we opted for segmentation from the outer retina to the choriocapillaris (ORCC: 0 µm from the outer plexiform layer to 49 µm underneath Bruch’s membrane). Because the morphological alterations in the retina often caused incorrect segmentation, all B-scans depicting the MNV were checked, and if necessary, segmentation lines were manually corrected.

**Figure 1:**
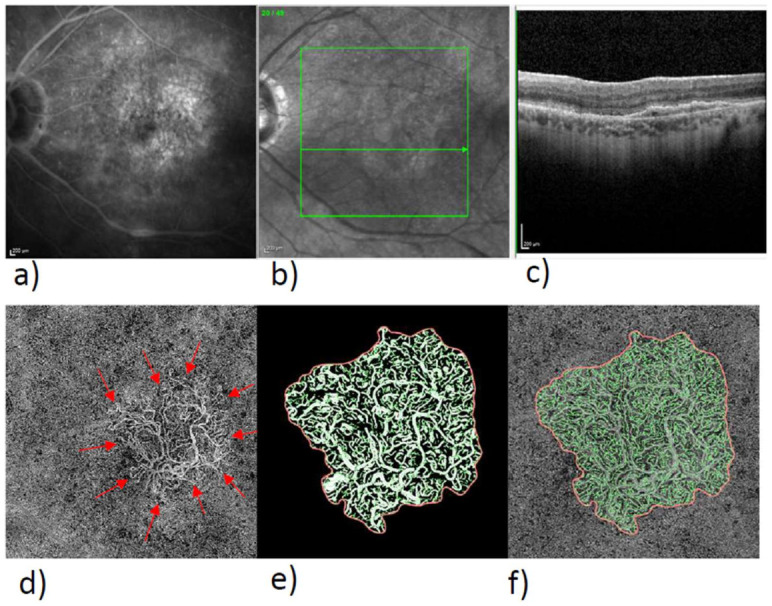
Multimodality imaging of a type 1 MNV in FA, OCT, and OCTA with skeletonized MNV. Overview of type 1 MNV in FA (**a**), near-infrared (NIR) image with line (**b**) of SD-OCT-B-Scan (**c**), en-face view of the ORCC slab (**d**), the red arrows mark the outer boundary of the MNV, enlarged view of binarized MNV (**e**), and skeletonized MNV (**f**).

**Figure 2:**
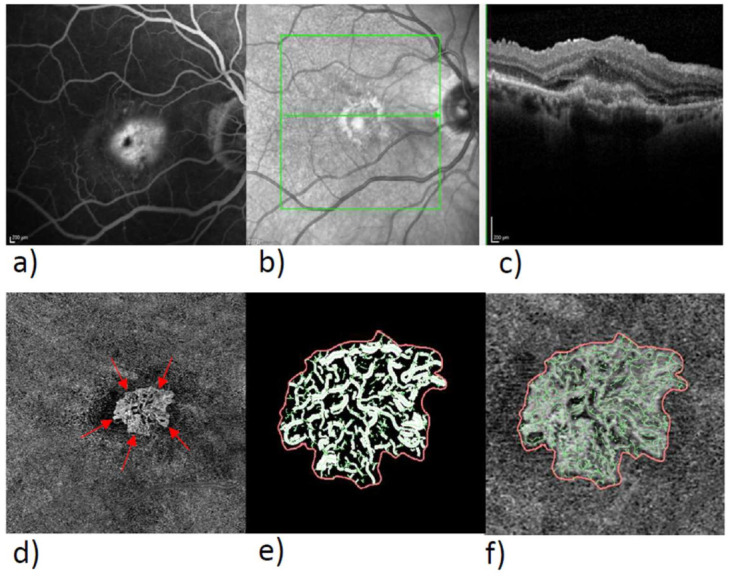
Multimodality imaging of a type 2 MNV in FA, OCT, and OCTA with skeletonized MNV. Overview of type 2 MNV in FA (**a**), NIR image with line (**b**) of SD-OCT (**c**), en-face view of the ORCC slab (**d**), the red arrows mark the outer boundary of the MNV, enlarged view of binarized MNV (**e**), and skeletonized MNV (**f**).

**Figure 3:**
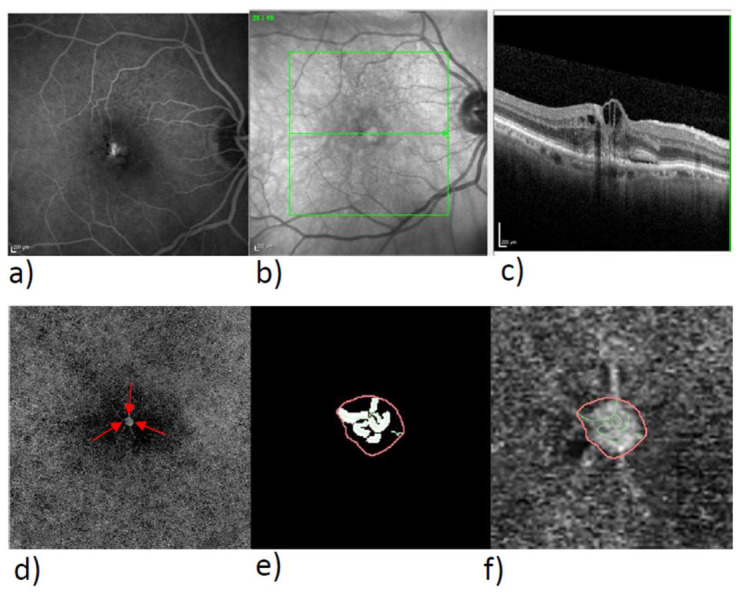
Multimodality imaging of a type 3 MNV in FA, OCT, and OCTA with skeletonized MNV. Overview of type 3 MNV in FA (**a**), NIR image with line (**b**) of SD-OCT (**c**), en-face view of the ORCC slab (**d**), the red arrows mark the outer boundary of the MNV, enlarged view of binarized MNV (**e**), and skeletonized MNV (**f**).

In the exported en-face OCTA images, we delineated the MNV by means of the program Fiji (National Institute of Mental Health, Bethesda, MD, USA), isolated the MNV from the rest of the image, and stored them for subsequent analysis. The vascular network of the MNV was extracted with the aid of MatLab (Mathworks, Natick, MA, USA Version R2014b). Skeletonization of the vasculature was achieved by multiscale calculation of the gradient field in the en-face OCTA image. This detected both thin and thick vascular segments as continuous midlines [15,18]. Next, the diameter of each vascular segment was determined so that segmentation of the vascular network could be calculated in addition to skeletonization. The borders of the vascular graphs represent the individual vascular segments, while the nodes represent the places where the vessels fork. The following six parameters were chosen for morphological characterization of the MNV: area, flow, fractal dimension (FD), total vascular length (sumL), number of vascular nodes (numN), and average vessel caliber (avgW). The area and sumL describe the MNV in terms of size and extent. The vascular parameters numN and avgW and FD directly describe the architecture of the MNV and provide information about the degree of branching and the maturity of the MNV. The FD, on the other hand, describes the overall complexity of a structure as a mathematical measure and has, in previous studies, been identified as a significant biomarker alongside anti-VEGF-receptor therapy. The flow is a measure of the proportion of vascularization of the MNV. It can be indicated by practically all commercially available OCTA devices, so its analysis can be done by anyone without complex image processing. Typical FA, SD-OCT, and OCTA images of type 1, 2, and 3 MNV are shown in Figure 1, Figure 2 and Figure 3, together with binarized and skeletonized views.

Patients with inadequate image quality (quality score < 6), retinal pathology other than nAMD, fibrosis, or GA were excluded. Unfortunately, patients with disturbing artifacts or no detectable MNV in OCTA could not be included.

### 2.3. Statistics

Data were analyzed using the statistics program R (Version 4.0.2). A significance level of 5% was used for all analyses. We check the variance homogeneity (Levene test) for comparison of type 1, 2, and 3 MNV and the normal distribution of the residuals (Shapiro–Wilk test). In cases our experiments violated these ANOVA preconditions, we applied the Kruskal–Wallis test. In a post hoc analysis, the Dunn test was performed for pairwise comparison, and *p*-values were adjusted using Bonferroni comparison.

## 3. Results

### 3.1. Demographic Data

We assessed 169 eyes of 158 treatment-naïve patients with MNV in nAMD. Sixty-one eyes were excluded due to insufficient signal strength for precise quantification, incomplete visualization of the MNV, and distorting artifacts. In further 18 eyes, no MNV could be delineated despite high image quality. Hence, the sensitivity of OCTA for the detection of MNV in our cohort was 83.3%.

Ninety eyes remained for further analysis. The mean age was 78.1 ± 6.9 years, and the mean best-corrected visual acuity (BCVA) was 0.59 ± 0.33 logMAR. As classified on FA and SD-OCT, 39 MNV were type 1, 32 were type 2, and 19 were type 3. An overview of the descriptive results from our study population with minimum, 1st quartile, median, 3rd quartile, maximum, mean, and standard deviation (SD) for the analyzed vascular parameters area, FD, numN, flow, sumL, and avgW are shown in Table 1.

Separated by types of MNV, the group of type 1 was on average 80.03 ± 6.41 years old, 25 were female, and 14 were male. The mean BCVA was 0.59 ± 0.33 logMAR. The mean age of the group of type 2 was 78.41 ± 6.66 years old, 18 were female, and 14 were male. The mean BCVA was 0.56 ± 0.32 logMAR. The mean age of the group of type 3 was 77.78 ± 6.48 years old, 12 were female, and 7 were male. The mean BCVA was 0.59 ± 0.34.

### 3.2. OCTA Variable Analysis

**Figure 4:**
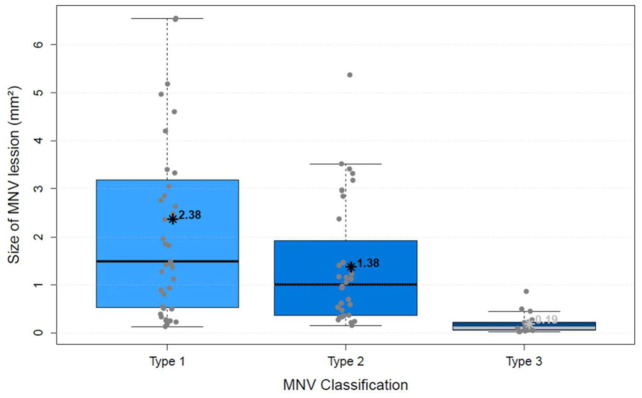
Distribution of size of MNV lesion in mm2 (area) of types 1, 2, and 3 MNV shown in boxplots. The box contains the measured values between the upper and lower quartile. All individual measured values are shown as dots. The whiskers visualize the last measured value that lies within 1.5 times the interquartile range; * = mean value, bar = median; Kruskal–Wallis test for group comparison *p* < 0.00001; Dunn test for post hoc analysis: MNV1 vs. MNV2 (*p* > 0.05), MNV1 vs. MNV3 (*p* < 0.00001), MNV2 vs. MNV3 (*p* < 0.00001).

**Figure 5:**
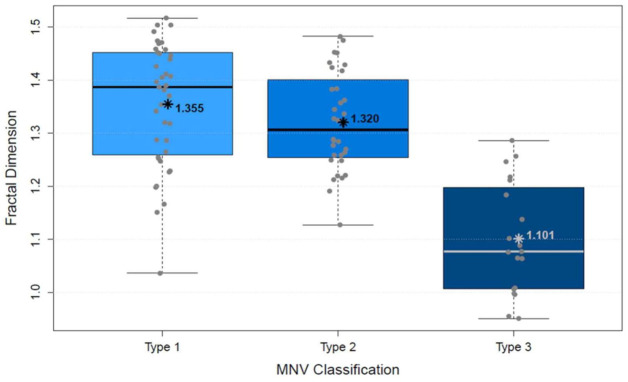
Distribution of fractal dimension of types 1, 2, and 3 MNV shown in boxplots. The box contains the measured values between the upper and lower quartile. All individual measured values are shown as dots. The whiskers visualize the last measured value that lies within 1.5 times the interquartile range; * = mean value, bar = median; Kruskal–Wallis test for group comparison *p* < 0.00001; Dunn test for post hoc analysis: MNV1 vs. MNV2 (*p* > 0.05), MNV1 vs. MNV3 (*p* < 0.00001), MNV2 vs. MNV3 (*p* < 0.00001).

**Figure 6:**
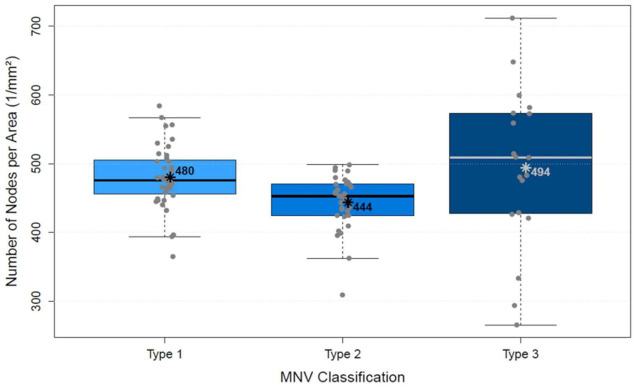
Distribution of number of nodes per area of types 1, 2, and 3 MNV shown in boxplots. The box contains the measured values between the upper and lower quartile. All individual measured values are shown as dots. The whiskers visualize the last measured value that lies within 1.5 times the interquartile range; * = mean value, bar = median; Kruskal–Wallis test for group comparison *p* < 0.005; Dunn test for post hoc analysis: MNV1 vs. MNV2 (*p* < 0.005), MNV1 vs. MNV3 (*p* > 0.05), MNV2 vs. MNV3 (*p* < 0.005).

**Figure 7:**
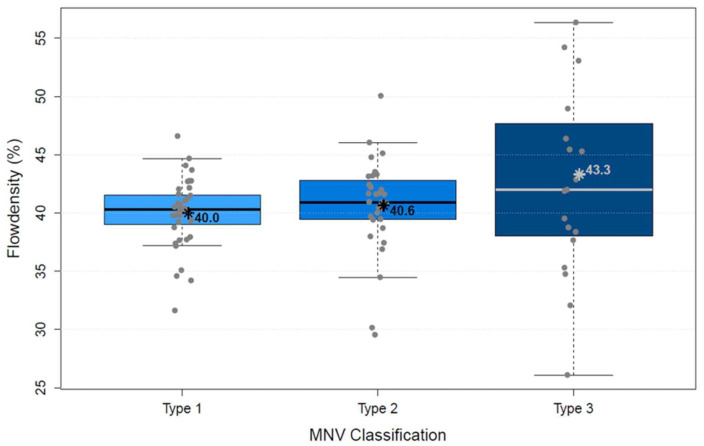
Distribution of flowdensity of types 1, 2, and 3 MNV shown in boxplots. The box contains the measured values between the upper and lower quartile. All individual measured values are shown as dots. The whiskers visualize the last measured value that lies within 1.5 times the interquartile range; * = mean value, bar = median; Kruskal–Wallis test for group comparison *p* > 0.05; Dunn test for post hoc analysis: MNV1 vs. MNV2 (*p* > 0.05), MNV1 vs. MNV3 (*p* > 0.05), MNV2 vs. MNV3 (*p* > 0.05).

**Figure 8:**
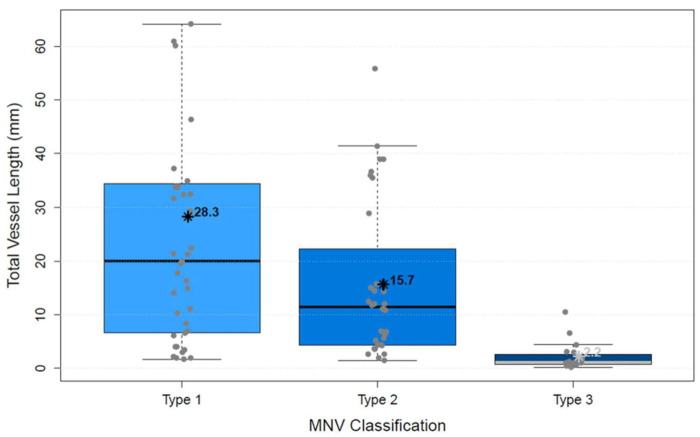
Distribution of total vessel length of types 1, 2, and 3 MNV shown in boxplots. The box contains the measured values between the upper and lower quartile. All individual measured values are shown as dots. The whiskers visualize the last measured value that lies within 1.5 times the interquartile range; * = mean value, bar = median; Kruskal–Wallis test for group comparison *p* < 0.00001; Dunn test for post hoc analysis: MNV1 vs. MNV2 (*p* > 0.05), MNV1 vs. MNV3 (*p* < 0.00001), MNV2 vs. MNV3 (*p* < 0.00001).

**Figure 9:**
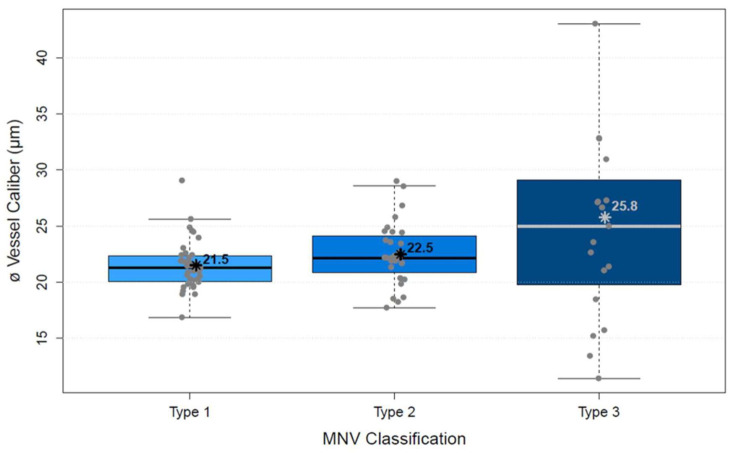
Distribution of average vessel caliber of types 1, 2, and 3 MNV shown in boxplots. The box contains the measured values between the upper and lower quartile. All individual measured values are shown as dots. The whiskers visualize the last measured value that lies within 1.5 times the interquartile range; * = mean value, bar = median; Kruskal–Wallis test for group comparison *p* < 0.00001; Dunn test for post hoc analysis: MNV1 vs. MNV2 (*p* > 0.05), MNV1 vs. MNV3 (*p* < 0.05), MNV2 vs. MNV3 (*p* > 0.05).

The analysis of vascular parameters of the MNV using en-face OCTA imaging showed the following results: type 1 and type 2 were similar as regards their area. The area of type 3 was significantly smaller than both type 1 and type 2 (*p* < 0.00001; Figure 4). FD showed no difference between types 1 and 2, but type 3 was significantly smaller than type 1 (*p* < 0.00001) and type 2 (*p* < 0.00001; Figure 5), which suggests a more complex vasculature in type 1 and 2. The numN differed significantly between types 1 and 2 and between types 2 and 3 for it being lower in type 2 in both comparisons, but we did not see a significant difference between types 1 and 3 (Figure 6). Additionally, there were no significant differences in flow density among the three types of MNV (Figure 7). As for sumL, types 1 and 2 showed no difference, but type 3 differed significantly from both type 1 (*p* < 0.00001) and type 2 (*p* < 0.00001; Figure 8) in regards of a lower sumL. There was no significant difference in avgW between types 1 and 2 or between types 2 and 3, but type 1 had a significantly lower avgW than type 3 (Figure 9).

The investigated vascular parameters show significant differences, especially between types 1/2 and type 3. Between type 1 and type 2, a significant difference could only be found for numN. No significant difference was found for flow between all types.

## 4. Discussion

MNV leads to a breakdown of the avascular retina and causes reduced phototransduction via displacement and disruption of photoreceptors. Many factors are involved in the development of MNV, one mechanism being the stimulation of vessel growth by the protein vascular endothelial growth factor A (VEGF-A). One protein that inhibits new blood vessels is sFLT-1, which is produced by cells in the photoreceptor layer and which is reduced in patients with AMD causing blood vessels to grow into the photoreceptor layer [19]. Other diseases that are based on instability of the photoreceptors, e.g., due to gene mutations in the peripherin-2 (PRPH-2) gene such as retinitis pigmentosa, can also lead to the formation of choroidal neovascularizations [20,21].

Currently, the vascular morphology of MNV in nAMD is not considered within the classification or assessment of the disease. In our study, we have qualitatively described and quantified morphological differences among treatment-naïve types 1, 2, and 3 MNV. OCTA imaging allows detailed analysis of the pathologic vasculature of MNV—in contrast to FA, where differentiation of vascular structures is particularly impeded by leakage. We believe that this is useful, especially in regard to the impending implication of artificial intelligence for automated analysis. Several studies have described the vascular morphology of MNV [8,9,10,22], although these descriptions include subjective assessments of the manifestation of MNV. In earlier research, we showed that a detailed mathematical description of vasculature is feasible and that changes in these parameters can be detected alongside anti-VEGF-receptor treatment [12,15]. Other authors have used OCTA for detailed quantification of vascular morphology in other retinal diseases, such as macular telangiectasia [23], diabetic retinopathy [24], retinal vascular occlusions [25], and glaucoma [26]. This shows that OCTA has considerable potential as it gives additional information for diagnosis and monitoring of various diseases and can be used as an adjunct to FA and SD-OCT.

Phasukkijwatana et al. found that type 3 MNVs were significantly smaller in area than type 1, which agrees with our results; furthermore, the difference in area between types 2 and 3 was also significant in our study [27]. We found no difference in this regard between type 1 and type 2, though it has to be kept in mind that detection of type 1 is less reliable due to signal attenuation by the RPE, whereby type 2 lies above the RPE.

Most of the significant differences in vascular morphology (area, FD, sumL, numN) were shown between types 1 and 3 and between types 2 and 3. This can be explained by the similar pathogenesis of types 1 and 2, which both arise in the choriocapillaris and grow towards the retina; in contrast, type 3 arise in the deep capillary plexus [28]. Typically, type 3 MNVs clinically go along with intraretinal spot hemorrhages outside the foveal zone, cystoid spaces, and development of a PED. They have a smaller area, lower sumL, and lower FD, but higher numN. This characteristic high-flow, tuft-shaped proliferation corresponds to the familiar findings of other OCTA analyses and to known patterns on ICG imaging representing descending vessels [29]. However, due to the direction of growth of neovascularization from the deep retinal vascular plexus towards the RPE, axial blood flow cannot be adequately detected by OCTA devices. This could also explain the lower sumL for type 3, as not all vascular segments are recorded.

Arrigo et al. also studied the vasculature of treatment-naïve MNV in nAMD [30]. They used the parameter “VDisp”, which represented the disorganization of the pathologic vasculature. They found a higher value in type 2 compared to type 1 and saw a possible explanation in the greater spread of type 2 above the RPE. This parameter cannot directly be compared with parameters obtained in our study, although shown significant differences between type 1 and type 2 in terms of numN could likewise indicate that type 1, limited by Bruch’s membrane, tend to extend more horizontally, while type 2 grow vertically through the RPE into the subretinal space. The higher number of bifurcations in type 1 could, however, be attributable to choriocapillary vasculature possibly included in segmentation in the presence of defects in Bruch’s membrane.

Nakano et al. also investigated the vascular morphology of types 1 and 2 MNV in detail and found lower junction densities in type 1 [10]. In our study, we found a higher density of vascular nodes in type 1—and also type 3—than in type 2. The results are not quite comparable because Nakano et al. calculated the number of nodes per unit of vascular length, while we counted the nodes per unit of MNV area.

The flow represents the proportion of all pixels that generate a detectable signal in the imaging window. In our study, the flow was the only one of the acquired parameters that showed no significant differences among the three types of MNV, which is in accordance with the findings of Arrigo et al. [30]. Other studies, however, have shown that flow decreases on the transition from the active to the inactive state after anti-VEGF-receptor therapy and thus may constitute an important parameter in monitoring the outcome of treatment [22,31].

Currently, there is no additional therapeutic or prognostic value in observing and analyzing MNV in detail. Nevertheless, we believe that it will have additional value in the future, as nAMD is a vascular disease and big differences in the vasculature of MNV are evident. For example, an artificial intelligence study also demonstrated that the en-face OCTA view of MNV in nAMD is more predictive than SD-OCT images for activity assessment [32]. This contradicts our current human assessment for the disease and shows that there are crucial vascular parameters of MNV that have not yet been identified. The analysis of the vasculature could also help to more accurately differentiate MNV types and, thus, improve diagnosis. Additionally, a new classification based on OCTA parameters could be implemented if correlations of these parameters with clinical data and morphological retinal findings were found in future works. In a recent pilot study [33], we have observed that, regardless of MNV type, patients with a smaller MNV area and sumL and higher flow had a significantly better visual outcome after one year of treatment. Likewise, higher flow and larger vessel caliber of MNV were significantly associated with fewer anti-VEGF injections needed in the first year of treatment. Of course, further comprehensive studies are necessary for validation. In addition, the vasculature of MNV could be used to better differentiate vascular changes alongside therapy and, thus, monitor therapeutic effects. Especially with regard to the development of new drugs with effects on other angiogenic signaling pathways, such as platelet-derived growth factor (PDGF) and Angiopoietin-1 (Ang1), proper interpretation of vascular morphology could provide important insights [34].

Our study has some limitations. Only 6 × 6 images were analyzed because larger MNVs are often not visualized entirely on 3 × 3 images. However, the lower resolution of 6 × 6 images means that fewer details can be discerned [35]. Moreover, the OCTA technique is subject to limiting factors such as shadow artifacts caused by corneal opacities, advanced cataract, retinal hemorrhage, retinal pigment epithelium, and all structures of the vitreous body and retina. Furthermore, only the blood flow in a predetermined span of time can be optically depicted by OCTA, and variable interscan time analysis (VISTA) has shown different patterns of vascular morphology at different moments in time [36]; therefore, we cannot be sure to what extend the vasculature visualized by OCTA corresponds to the actual vascular structures.

## 5. Conclusions

In summary, OCTA is capable of providing detailed information on the vascular morphology of patients with MNV in nAMD; above all, it shows the differences between types 1/2 and type 3 MNV. These data will be of great interest in the future for proper monitoring of the disease and to assess the efficacy of medication, particularly with regard to new substances, and further research should be devoted to this topic.

## Figures and Tables

**Table 1:** Overview of the analyzed vascular parameters for the whole sample and for each MNV type.

Variable	Classification	Minimum	1. Quartil	Median	3. Quartil	Maximum	Mean	SD
area in mm^2^	total	0.02	0.26	0.87	2.37	9.51	1.56	1.9
area in mm^2^	type 1	0.13	0.53	1.49	3.18	9.51	2.38	2.33
area in mm^2^	type 2	0.15	0.36	1.01	1.7	5.37	1.38	1.3
area in mm^2^	type 3	0.02	0.06	0.1	0.22	0.86	0.19	0.21
FD	total	0.95	1.21	1.29	1.41	1.52	1.29	0.14
FD	type 1	1.04	1.26	1.39	1.45	1.52	1.35	0.12
FD	type 2	1.13	1.26	1.31	1.39	1.48	1.32	0.09
FD	type 3	0.95	1.01	1.08	1.2	1.29	1.1	0.11
numN 1/mm^2^	total	265.31	437.36	469.55	497.59	712	470	67.82
numN 1/mm^2^	type 1	365.04	455.96	475.64	505.64	584.17	480	46.06
numN 1/mm^2^	type 2	309.03	424.84	452.67	470.42	498.59	444	40.3
numN 1/mm^2^	type 3	265.31	427.54	508.77	572.97	712	494	115.7
Flowdensity	total	26.1	38.76	40.77	42.75	64.03	40.9	5.3
Flowdensity	type 1	31.1	38.99	40.31	41.53	46.59	40	2.94
Flowdensity	type 2	29.53	39.43	40.9	42.6	50.07	40.6	4.04
Flowdensity	type 3	26.1	38.03	41.99	47.68	64.03	43.3	9.18
sumL in mm	total	0.2	2.94	10.36	29.32	107.44	18.3	22.55
sumL in mm	type 1	1.66	6.7	20	34.3	107.44	28.3	27.8
sumL in mm	type 2	1.47	4.38	11.45	18.99	55.76	15.7	14.72
sumL in mm	type 3	0.2	0.7	1.08	2.61	10.48	2.21	2.57
avgW in µm	total	11.42	20.26	21.88	24.32	54.11	22.8	5.37
avgW in µm	type 1	16.83	20.07	21.29	22.31	29.05	21.5	2.2
avgW in µm	type 2	17.69	21.09	22.13	23.92	29.02	22.5	2.72
avgW in µm	type 3	11.42	19.76	24.96	29.1	54.11	25.8	10.32

FD—fractal dimension; numN—number of vascular nodes; sumL—total vascular length; avgW—average vessel caliber; SD—standard deviation.

## Data Availability

All data used to support the findings of this study are included within the article and are available from the corresponding author upon reasonable request.

## Abbreviations

avgW	Average vessel caliber
CNV	Choroidal neovascularization
FA	Fluorescence angiography
FD	Fractal dimension
ICG	Indocyanine green
MNV	Macular neovascularization
nAMD	Neovascular age-related macular degeneration
numN	Number of vascular nodes
OCTA	Optical coherence tomography angiography
PED	Pigment epithelial detachments
RPE	Pigment epithelium
SD	Standard deviation
SD-OCT	Spectral-domain optical coherence tomography
sumL	Total vascular length
